# Recurrent Pyogenic Cholangiohepatitis: A Case Report

**DOI:** 10.7759/cureus.59142

**Published:** 2024-04-27

**Authors:** Wajd Y Assahafi, Basil M Sindy, Yaser S Alqahtani, Abdulaziz H Albarakati, Wael Z Hossameldin

**Affiliations:** 1 Internal Medicine, AlNoor Specialist Hospital, Makkah, SAU; 2 Gastroenterology, AlNoor Specialist Hospital, Makkah, SAU

**Keywords:** liver abscess, cholangiohepatitis, pyogenic, recurrent, recurrent pyogenic cholangiohepatitis

## Abstract

Recurrent pyogenic cholangitis (RPC), which is most commonly seen in Asian populations, is characterized by strictures and dilatation of both intrahepatic and extrahepatic bile ducts, along with the formation of pigmented stones inside the ducts. The most common symptoms are recurrent right upper quadrant pain, jaundice, and fever. Additionally, leukocytosis and elevated alkaline phosphatase and bilirubin levels may also be present.

We report the case of a 43-year-old Bangladeshi male patient with a medical background of chronic hepatitis B infection and recurrent liver abscesses who presented to the emergency department with abdominal pain and fever lasting for two days.

Given the clinical context of our patient, a diagnosis of RPC was made, and the patient was referred to a higher-level center for further management. Our case highlights the importance of considering RPC as part of the differential diagnosis in patients presenting with recurrent liver abscesses and features of ascending cholangitis.

## Introduction

Recurrent pyogenic cholangiohepatitis (RPC), also known as oriental cholangiohepatitis, is a condition marked by recurrent bacterial cholangitis, biliary strictures, and hepatolithiasis [1,2]. The term oriental cholangiohepatitis was first used by Digby in 1930 to describe a type of cholangiohepatitis observed in the Chinese population [2]. Although the etiology of RPC is uncertain, it has been associated with ethnic factors, dietary intake, and infection with bacterial and parasitic pathogens, leading to anatomical anomalies in the bile ducts and secondary stone formation [1-3]. These stones are typically composed of calcium bilirubinate and are found in the intrahepatic and extrahepatic bile ducts, but usually not in the gallbladder [4].

RPC typically presents with repeated attacks of fever, right upper abdominal pain, and jaundice (i.e., Charcot’s triad). These attacks can be complicated by abscess formation in the liver, which can result in septicemia or shock, characterized by mental confusion and hypotension (i.e., Reynold’s pentad). Other complications include acute biliary pancreatitis, obstructive jaundice, liver atrophy, and liver cirrhosis. Patients with RPC are also at a higher risk of cholangiocarcinoma [1,2,5,6].

Diagnosis of RPC is based on a compatible epidemiological background, clinical presentation, and radiological findings [2]. Characteristic radiological findings include intrahepatic biliary strictures and diffuse biliary dilatation with intraductal calculi [2,7]. Management of RPC requires an individualized and multidisciplinary approach, including antimicrobial therapy, endoscopic management, and possible surgical resection [2,3].

## Case presentation

A 43-year-old Bangladeshi male with a medical history of chronic hepatitis B infection complicated by liver cirrhosis and recurrent liver abscesses presented to the emergency department with abdominal pain and fever lasting two days. He described the pain as constant and dull in the right upper quadrant, which started gradually, had not radiated elsewhere, was aggravated by food, and was relieved by analgesia. He also reported undocumented on-and-off fever and yellowish discoloration of the eyes. He denied a history of nausea, vomiting, changes in bowel movements, or changes in stool or urine color. There was no history of weight loss, loss of appetite, night sweats, or fatigue. The patient had been hospitalized twice before with similar symptoms: once three years prior, and then again one year prior to presentation. During both hospitalizations, he was found to have a liver abscess that was managed by antibiotics and percutaneous drainage.

Upon examination in the ED, the patient appeared jaundiced and was in pain. He was hemodynamically stable with a body temperature of 37°C, a pulse rate of 98 bpm, a blood pressure of 121/75 mmHg, a respiratory rate of 18 breaths per minute, and an oxygen saturation of 98% on room air. An abdominal examination showed a symmetrical, nondistended abdomen, no stigmata of liver disease, mild tenderness of the right upper quadrant, and hepatomegaly 3-4 cm below the costal margin. Murphy's sign was negative, and no other findings were apparent.

Additionally, the patient had the following laboratory results (Table 1), an abdominal ultrasound showed an enlarged liver (19.2 cm) and a heterogeneous echogenic area in the right hepatic lobe most likely representing an abscess. The left hepatic lobe was not appreciated. The common bile duct was not dilated and measured 0.4 cm. There was no sonographic evidence of cholelithiasis, acute cholecystitis, or biliary obstruction.

**Table 1:** Summary of laboratory findings

	Patient’s results	Laboratory reference range
Hemoglobin (g/L)	112	120-150
Leukocytes: WBC count (10⁹/L)	26.6	4-11
Platelet count (10⁹/L)	142	150-400
Prothrombin time (sec)	19.5	11-16
Partial thrombin time (sec)	37.7	26-39
International normalized ratio (INR)	1.46	0.8-1.2
Alanine transaminase (U/L)	98	14-60
Aspartate transaminase (U/L)	61	15-37
Total bilirubin (µmol/L)	35	0-18.7
Direct bilirubin (µmol/L)	22	0-3
Amylase (U/L)	20	25-110
Creatinine (µmol/L)	78	44-90
Blood urea nitrogen (mmol/L)	4.8	2.6-6.4

**Figure 1:**
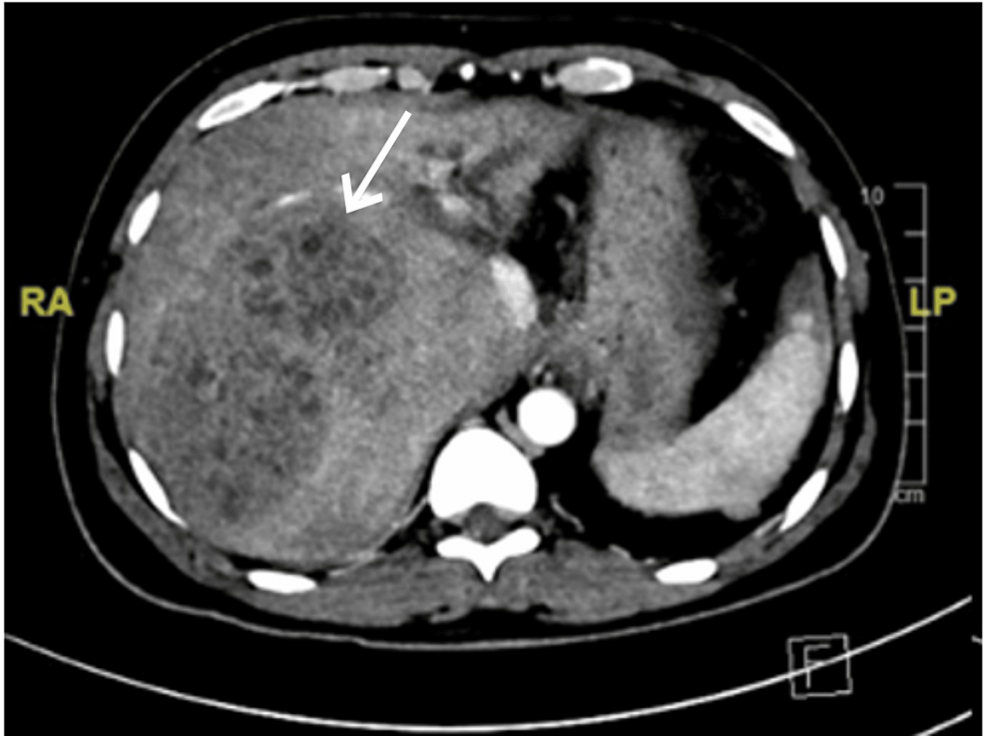
Axial computed tomography triphasic scan showing an enlarged liver (measuring 21 cm). A multiloculated complex cystic lesion was observed, representing an abscess measuring 9.7 x 6.7 x 10 cm in the anteroposterior, transverse, and craniocaudal dimensions occupying the right liver lobe, segments VII and VIII.

The patient was admitted to the general ward of the hospital with a case of recurrent liver abscess. Considering the diagnosis, he was started on cefotaxime and metronidazole empirically. Computed tomography (CT) triphasic scanning showed an enlarged liver with a normal smooth outline that was mildly compressing the left liver lobe and inferior vena cava with no obstruction. A multiloculated complex cystic lesion representing an abscess occupying the right liver lobe was noted. Other findings included abdominal free fluid, pleural effusion, and lower lobe consolidation (Figure 1). A 12-F drainage catheter was inserted under Ultrasound guidance, with purulent, greenish fluid discharge. Cultures from the drained fluid came back positive for *Klebsiella pneumoniae* carbapenem-resistant *Enterobacteriaceae* (CRE), and the patient was started on tigecycline for 14 days.

**Figure 2:**
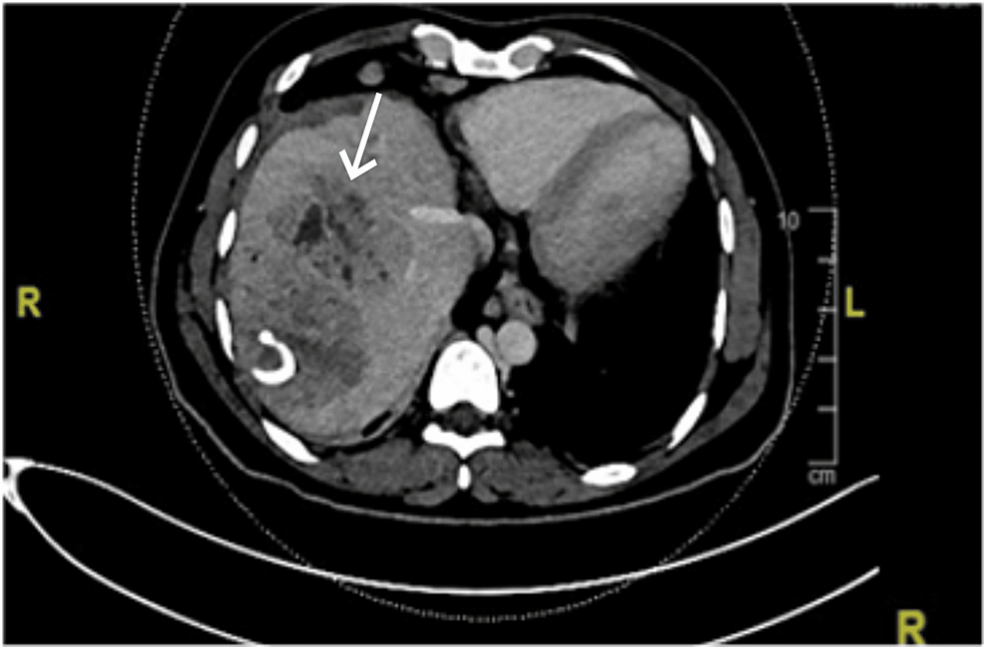
Axial computed tomography scan of the abdomen and pelvis showing an increase in the size of the previous abscess, with new abscess formation (3.7 x 4.6 x 6 cm), focal intrahepatic ductal dilation, and atrophic left liver lobe.

A repeated CT scan of the abdomen showed an increase in the size of the previous abscess, with new abscess formation, focal intrahepatic ductal dilation, and atrophic left liver lobe (Figure 2). 

**Figure 3:**
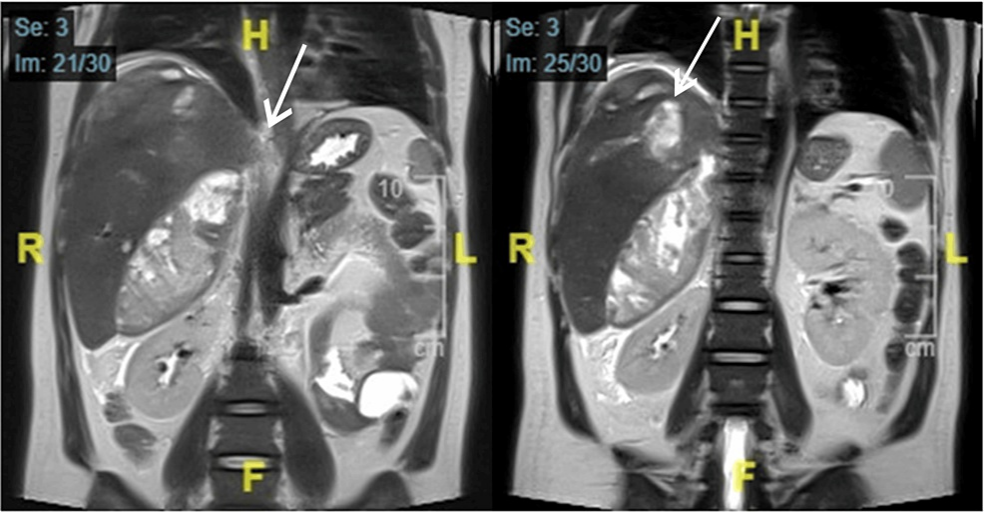
Coronal magnetic resonance imaging of the liver/magnetic resonance cholangiopancreatography showing a newly developed subcapsular collection. The left hepatic lobe was atrophied, with dilated bile ducts and multiple intraductal stones.

**Figure 4:**
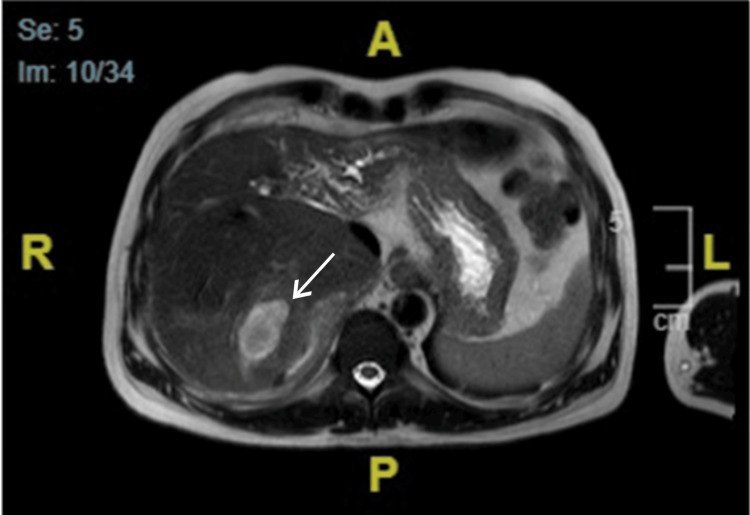
Axial magnetic resonance imaging of the liver. An ill-defined mass is seen in the right hepatic lobe showing high signal intensity in T2.

The infectious disease physician planned to stop tigecycline, and the patient was started on ceftazidime/avibactam for 10 days. Considering the possibility of oriental cholangiohepatitis, magnetic resonance imaging of the liver and magnetic resonance cholangiopancreatography (MRCP) was performed, both showing partial regression of the previous collection and a newly developed subcapsular collection. The left hepatic lobe was atrophied, with dilated bile ducts and multiple intraductal stones (Figures 3, 4). Given the patient’s medical history and imaging findings, he was diagnosed with oriental cholangiohepatitis and referred to a higher-level center for further management. However, the patient failed to follow up at our hospital.

## Discussion

RPC is a complex condition that is endemic in China, Japan, Taiwan, Vietnam, and Korea, and can be referred to as oriental cholangiohepatitis. The underlying mechanism of RPC remains unclear; it commonly presents with recurring bouts of Charcot’s triad (i.e., fever, jaundice, and right upper quadrant pain), in association with intrahepatic pigment stones and intrahepatic biliary obstruction, requiring repeated medical, radiological, and possibly surgical interventions. 

Due to its chronic nature, RPC patients are at a higher risk of complications such as acute biliary pancreatitis, obstructive jaundice, liver atrophy, cholangiocarcinoma, and liver cirrhosis, and may present with features suggestive of such conditions. These patients may also report a history of past procedures, including endoscopic drainage and endoscopic retrograde cholangiopancreatography (ERCP), as well as recurrent antibiotic use. 

The diagnosis of RPC depends on clinical history and, most importantly, imaging. Ultrasonography is the first line of investigation, and it is useful in evaluating and assessing the presence of other possible causes of cholangitis. Characteristic radiological findings include intrahepatic biliary strictures and diffuse biliary dilatation with intraductal calculi. These findings are better visualized with a CT scan of the abdomen. CT scans are also better at evaluating associated potential pathologies and are less operator-dependent. The gold-standard non-invasive modality, however, is MRCP, as it is superior to CT and US in evaluating and visualizing the characteristic intraductal calculi and bile duct strictures. 

An individualized and multidisciplinary approach is required when managing such a patient, which should include resuscitation with acute patients, antimicrobial therapy, endoscopic management, and possible biliary resection as a definitive treatment for those who do not respond to medical management. ERCP can be used to both diagnose and treat RPC. In cases of diffuse bilateral disease, liver transplantation could also be considered. On the other hand, the prognosis of RPC is not clear and well documented; however, it is related to many factors, such as the presence or absence of other co-morbidities, liver failure, or malignancy. 

To the best of our knowledge, this is the first case of RPC reported in Saudi Arabia. Our patient was a young Bangladeshi male who presented with recurrent liver abscesses requiring repeated admissions for antibiotics and drainage. The diagnosis was made based on the clinical context of our patient and radiological findings that were highly compatible with oriental cholangitis (Figures 1-4). Laboratory findings were nonspecific (Table 1), and other possible causes of cholangitis were ruled out. The patient was treated with antibiotics and percutaneous transhepatic drainage as an initial management for cholangitis and then referred to a hepatobiliary surgery center for further directed management and possible surgical intervention.

## Conclusions

RPC is a rare condition that is often overlooked in patients presenting with features of ascending cholangitis and other biliary conditions. A multidisciplinary team approach is essential for developing an individualized, targeted treatment plan, which can result in improved outcomes. We believe that understanding the underlying pathology is crucial to avoiding an incorrect diagnosis and that RPC should be considered a potential cause in patients with recurrent liver abscesses.
